# Factors related to health literacy among Brazilian adolescents: cross-sectional study

**DOI:** 10.1590/1980-220X-REEUSP-2023-0310en

**Published:** 2024-02-26

**Authors:** Sidiany Mendes Pimentel, Marla Andréia Garcia de Avila, Vinnicius Dias Alves de Medeiros, Rafaela Aparecida Prata, Hélio Rubens de Carvalho Nunes, Juliana Bastoni da Silva

**Affiliations:** 1 Universidade Federal do Tocantins, Pós-graduação em Ciências da Saúde, Palmas,TO, Brazil.; 2 Universidade Estadual Paulista “Júlio de Mesquita Filho”, Faculdade de Medicina de Botucatu, Botucatu, SP, Brazil.; 3 Universidade Federal do Tocantins, Graduação em Enfermagem, Palmas, TO, Brazil.

**Keywords:** Adolescent Health, Health Literacy, Health promotion, Social Determinants of Health, Adolescent Development, Salud del Adolescente, Alfabetización en Salud, Promoción de la Salud, Determinantes Sociales de la Salud, Desarrollo del Adolescente, Saúde do Adolescente, Letramento em Saúde, Promoção da Saúde, Determinantes Sociais da Saúde, Desenvolvimento do Adolescente

## Abstract

**Objective:**

To analyze the relationship between sociodemographic and clinical factors with health literacy in Brazilian adolescents.

**Method:**

This is a cross-sectional study with 526 adolescents aged 14 to 19. Data were collected virtually between July and September 2021 using a sociodemographic characterization questionnaire, clinical profile and the Health Literacy Assessment Tool – Portuguese version. The variables were evaluated by multiple linear regression with normal response, with significance p < 0.05.

**Results:**

The average age was 16.9 years (±1.6), the average health literacy score was 25.3 (±5.4). Female gender (p = 0.014), university educational level (p = 0.002) and use of medication (p = 0.020) were related to higher levels of health literacy. Adolescents with chronic illnesses had a higher total literacy score, on average 1.51 points, compared to those without chronic illnesses.

**Conclusion:**

Male adolescents and those with less education performed worse in health literacy and, therefore, deserve special attention in health promotion actions.

## RESUMO

**Objetivo:**

Analisar a relação entre fatores sociodemográficos e clínicos com o letramento em saúde de adolescentes brasileiros.

**Método:**

Trata-se de um estudo transversal com 526 adolescentes de 14 a 19 anos. Os dados foram coletados virtualmente entre julho e setembro de 2021 por meio de questionário de caracterização sociodemográfica, do perfil clínico e do Health Literacy Assessment Tool – versão em português. As variáveis foram avaliadas por regressão linear múltipla com resposta normal, com significância p < 0,05.

**Resultados:**

A média de idade foi de 16,9 anos (±1,6), a pontuação média do letramento em saúde foi de 25,3 (±5,4). Sexo feminino (p = 0,014), maior escolaridade (p = 0,002) e uso de medicamentos (p = 0,020) foram relacionados a maiores níveis de letramento em saúde. Adolescentes com doenças crônicas apresentaram pontuação total do letramento superior, em média 1,51 pontos, comparados aos sem doença crônica.

**Conclusão:**

Adolescentes do sexo masculino e os com menor escolaridade apresentaram pior desempenho no letramento em saúde e, por isso, merecem especial atenção nas ações de promoção da saúde.

## RESUMEN

**Objetivo:**

Analizar la relación entre factores sociodemográficos y clínicos con la alfabetización en salud de adolescentes brasileños.

**Método:**

Se trata de un estudio transversal llevado con 526 adolescentes entre 14 y 19 años. Los datos se recogieron virtualmente entre julio y septiembre de 2021 mediante un cuestionario de caracterización sociodemográfica, de perfil clínico y de la Health Literacy Assessment Tool – versión en portugués. Las variables se sopesaron por regresión linear múltiple con respuesta normal y significación p < 0,05.

**Resultados:**

La edad promedio era de 16,9 años (±1,6) y la puntuación media en alfabetización en salud de 25,3 (±5,4). El sexo femenino (p = 0,014), la mayor escolaridad (p = 0,002) y el uso de medicación (p = 0,020) estaban relacionados con niveles más altos de alfabetización en salud. Los adolescentes con enfermedades crónicas obtuvieron una puntuación total de alfabetización más alta, en media, 1,51 puntos, en comparación con los que no padecían dichas enfermedades.

**Conclusión:**

Adolescentes del sexo masculino y aquellos con menos escolaridad presentaron un desempeño pobre en la alfabetización en salud y, por esa razón, necesitan atención especial durante las acciones de promoción de la salud.

## INTRODUCTION

Health information and the ways in which it is shared with the community, through health professionals and organizations, are undergoing transformation, especially in younger populations, given the development of information technologies^(1)^. The interaction of individuals with health information is investigated by Health Literacy (HL), a field under construction that has been considered of great relevance and impact on the health of the population, being designated as a new vital sign and identified as a global goal to improve health promotion^(2)^. HL is defined by the World Health Organization (WHO)^(3)^ as the personal knowledge and skills accumulated throughout life that enable access, understanding, evaluation and use of health information and services to promote and maintain individual and collective health care.

HL can be understood as the observable result of actions aimed at promoting people’s empowerment and participation in their communities and healthcare^(2)^. HL has an interactive and intersectoral character, it is a modifiable social determinant of health and a critical element for development throughout life cycles, especially in adolescence. At this stage, HL is identified as a mediator in decision-making related to health and the adoption of protective behaviors^(4)^ by adolescents; a high level of HL can enable them to take control of their own health condition.

Adolescence is a phase of intense changes in the biopsychosocial areas, defended by professionals and researchers as a crucial stage for the development of actions aimed at promoting HL^(5)^. However, there is no bigger picture of HL conditions in different regions of Brazil, unlike countries such as China, Italy and Australia, which have already presented data showing positive aspects of HL in adolescents in their countries^(6–8)^.

Studies indicate that adolescents with better HL levels are more likely to present health-protective behaviors, to present more effective communication with health professionals and to better assess their own health status^(9)^.

In order to support HL research on children and young people, German researchers created a six-dimensional theoretical model. The proposal recognizes the specific characteristics of the groups and considers their specificities regarding HL in relation to adults. The first ‘D’, for dimension, deals with epidemiological differences: adolescence is a window of opportunity for promoting HL to interrupt the cycle of chronic diseases that are related to sedentary behaviors. The second ‘D’ addresses demographic patterns and inequalities, that is, economically disadvantaged environments reduce opportunities to promote and protect health and increase the impact of risk factors. In the third ‘D’, development and the socialization process are considered; In this dimension, the authors argue that HL can be a strong resource for developing coping and resilience strategies in the family, school and sociocultural environment. The fourth ‘D’ analyzes the adolescent’s relationship of dependence within power structures and intergenerational relationships, as well as their interface with the opinions and experiences developed as active subjects. In line with this, the fifth ‘D’ deals with citizen democracy and considers that adolescents should be encouraged to collaborate and get involved with HL in the community where they live, in primary health care actions and in school health. The sixth ‘D’ considers the digital world in which children and adolescents grow and develop, as well as its influence on HL^(10)^.

In Brazil, investigations into HL in adolescence are still scarce. However, there are studies with adolescents in the country that deal with relevant topics such as the association of HL and contraceptive methods^(11)^; functional health literacy, quality of life and contexts of violence^(12)^ and HL, assessment of the threat of COVID-19 and vaccination intention^(13)^.

HL has been identified as fundamental for training and allowing adolescents to engage in health promotion actions, which could contribute to positively influencing other social determinants of health^(3)^. In this sense, this research aimed to analyze the relationship between sociodemographic and clinical factors with the health literacy of Brazilian adolescents.

## METHOD

### Study Design

This is a cross-sectional study, carried out with adolescents living in Brazil, with data collection in a virtual environment. The population was made up of Brazilian adolescents in middle and late adolescence, aged between 14 and 19 years old^(14)^. Data communication follows the recommendations of the Strengthening the Reporting of Observational Studies in Epidemiology (STROBE)^(15)^ statement. This research derives from a multivariate study called “Health literacy, threat from COVID-19 and vaccination intention of Brazilian adolescents”^(13)^.

### Setting, Sample and Data Collection Procedures

The research covered five Brazilian macro-regions, whose estimated population of adolescents is 18.452.517^(16)^. Data collection took place in a virtual environment using the Google Forms tool. Participants who agreed to the terms of consent/assent were included. Forms that failed to be completed were considered lost.

The sample for this study resulted from the sample calculation carried out for the matrix project, considering: simple random sampling; previous studies on the topic^(17,18)^; type I and II errors, defined as equal to 0.05 and 0.10, respectively; comparison between two binomial proportions coming from independent samples; as well as the presence of nine confounding variables, with the addition of 15 subjects for each of them, inserted in the multiple regression model, which resulted in 526 adolescents. Data collection was carried out from July 13^th^ to September 30^th^, 2021; Therefore, 528 forms were received and 2 were considered losses due to failure to complete. The recruitment of adolescents occurred, predominantly, using the snowball technique, a method in which the participants themselves share the forms with their peers. For publicity, posters and videos were produced and distributed on social networks (Instagram, Facebook, Twitter, TikTok and Kwai), on communication platforms (WhatsApp, Gmail) and phone calls were also made. Institutions such as schools, universities, churches, municipal and state health and education departments were invited to share the invitation for the research and recruit participants, in the five regions of Brazil.

### Research Instruments

The research form consisted of 16 items and collected sociodemographic data, clinical profile and HL of the adolescents. The sociodemographic and clinician profile variables were collected using a specific instrument, developed by the authors, based on social indicators from the Brazilian Institute of Geography and Statistics – IBGE^(19)^. This part was structured with eight items that investigated information regarding state of residence, age, gender, income, education, history of illnesses, hospitalizations and use of medications in the last six months.

Data regarding HL were collected using the Health Literacy Assessment Tool – health literacy questionnaire, Portuguese version (p-HLAT-8), translated and validated in Brazil^(17)^. The HLAT-8 was developed and tested in Switzerland with the aim of investigating different dimensions of the HL of young people in the socio-family context^(18)^. The Brazilian version (p-HLAT-8) was applied to 472 Brazilian university students and demonstrated reliability for calculating the general score on the HL considering the due weight for each item, with Cronbach’s alpha 0.74.

The p-HLAT-8 has eight questions with answers on a Likert scale that ranges from zero to a maximum of five points. The items assess: (i) understanding of health information (questions 1 and 2) which total 10 points; (ii) search for health information (questions 3 and 4) which total 8 points; (iii) health interactivity (questions 5 and 6) which total 10 points; and (iv) critical health knowledge (questions 7 and 8) which total 9 points. The overall score varies from zero (worst score) to 37 points (best/ideal score), with no cut-off point or classification of HL as low, moderate or high level; it is considered that the higher the score for the aforementioned instrument, the higher the HL of the research participant.

### Data Analysis

Descriptive statistics were used for sociodemographic and clinical characterization. To verify associations between HL and independent variables, simple regression models with normal response were used. The variables that showed an association with p < 0.10 were taken to a multiple linear regression model, with a normal response. In the multiple linear regression models, relationships that presented p < 0.05 were considered statistically significant.

### Ethical Aspects

The study was approved by the Research Ethics Committee of the Federal University of Tocantins (UFT), under number 4.833.554/2021, in accordance with National Health Council Resolution No. 466/2012^(20)^. The Free and Informed Consent Form (FICF) was used for participants over 18 years old and for the parents/guardians of those under 18 years old. Minors under 18 were asked to accept the Assent Form.

## RESULTS

Five hundred and twenty-six Brazilian teenagers participated in the research, from 25 states in the country and the Federal District. Only the state of Amazonas was not represented in this study. The adolescents had a mean age of 16.9 years (±1.6); of these, 40.7% (n = 214) were in middle adolescence and 59.3% (n = 312) were in late adolescence. It stands out from Table 1 that 67.7% (n = 356) of the participants were female, 67.1% (n = 353) were studying or had completed high school and 49.0% lived in the North Region (n = 258).

**Table 1:** Sociodemographic characterization and clinical profile of adolescents from the five regions of Brazil (n = 526) – Palmas, TO, Brazil, 2021.

Variable	Total	
	N	%
**Gender**		
Female	356	67.7
Male	170	32.3

**Age (in Years)**		
14	49	9.3
15	71	13.5
16	94	17.9
17	71	13.5
18	137	26.0
19	104	19.8

**Region**		
North	258	49.0
North East	31	5.9
Midwest	36	6.8
Southeast	191	36.3
South	10	1.9

**Education (in progress or completed)**		
Elementary School	67	12.7
High school	353	67.1
University education	106	20.2

Income (minimum wage)*		
< 0.5	116	22.1
0.5 – <1	52	9.9
1 – <2	136	25.9
2 – <5	141	26.8
5 – <10	45	8.6
10 – <20	26	4.9
≥20	10	1.9

**Chronic disease**		
No	477	90.7
Yes	49	9.3

**Recent hospitalization**		
No	505	96.0
Yes	21	4.0

Medication use		
No	424	80.6
Yes	102	19.4

*Minimum wage in 2021: R$ 1,212^1^

Source: Research data.

The states with the most participants were: Tocantins with 46.0% (n = 242), São Paulo with 28.5% (n = 150), Minas Gerais with 4.9% (n = 26) and Goiás with 3.8% (n = 20).

Regarding HL, the average score found was 25.3 points (±5.4), with a median of 26.0 (minimum of 0 and maximum of 37). The bivariate analysis is detailed in Table 2, which highlights that male adolescents had a lower HL total score than female adolescents (p = .010). It is also observed that adolescents attending higher education had higher scores than those with basic education, on average 2.70 points higher. The total HL score of adolescents who reported living with a chronic illness was, on average, 1.51 points higher when compared to participants without this type of health problem. Regarding the use of medications, HL score was higher (1.87 points on average) among those who used any medication when compared to those who did not use it.

**Table 2:** Bivariate analysis of sociodemographic and clinical factors related to health literacy among adolescents from the five regions of Brazil) – Palmas, TO, Brazil, 2021.

Variable	B	CI95%		p*
**Male gender**	−1.30	−2.29	−0.32	**.010**
**Brazil Regions**				
South region	0.77	−2.66	4.19	.662
Southeast region	0.75	−0.27	1.76	.150
Midwest region	−0.48	−2.37	1.41	.620
North East region	0.61	−1.41	2.63	.554
North East**	0a			
Age***	0.23	−0.06	0.52	.120
**Educational background**				
Universtiy level (in progress)	2.70	1.06	4.34	**.001**
High school level (in progress or completed)	0.60	−0.80	2.01	.398
Elementary school level (in progress or completed)****	0a			
**Income**	−0.18	−3.68	3.32	.919
**Income of more than twenty minimum wages**				
Income between ten and twenty minimum wages	1.40	−0.91	3.70	.235
Income between five and ten minimum wages	1.06	−0.80	2.93	.264
Income between two and five minimum wages	−0.09	−1.42	1.24	.896
Income between one and two minimum wages	0.19	−1.16	1.53	.785
Income between half and one minimum wage	−0.39	−2.17	1.38	.664
Income up to half the minimum wage*****	0a			
**Chronic illness^†^**	1.51	−0.08	3.11	**.063**
**Recent hospitalization^†^**	−0.83	−3.21	1.54	.492
**Medication use^†^**	1.87	0.71	3.04	**.002**

B: Estimation of regression model parameters; CI: confidence interval; *Regression with normal response; **Reference for regions of Brazil; ***Continuous quantitative variable; ****Reference for education levels; *****Reference for income levels; †Dichotomous variables, had as reference the absence of the analyzed event.

Source: Research data.

Table 3 presents the multivariate analysis between the HL and the variables investigated, using multiple linear regression. There is evidence of an association between lower HL scores and males, when compared to females (p = 0.014); the results also show the association between higher HL scores and educational level (p = 0.002), as well as the use of medications (p = 0.020).

**Table 3:** Multivariate analysis of sociodemographic and clinical factors related to health literacy among adolescents from the five regions of Brazil (n = 526) – Palmas, TO, Brazil, 2021.

Variable*	B	CI95%		p**
Intercept	24.48	23.17	25.78	0.000
**Male gender**	−1.22	−2.19	−0.25	**.014**
**Educational background**				
Universtiy level (in progress)	2.55	0.93	4.18	**.002**
High school level (in progress or completed)	0.57	−0.82	1.96	.419
Elementary school level (in progress or completed)****	0a			
**Chronic illness†**	0.68	−0.96	2.33	.417
**Medication use†**	1.44	0.23	2.65	**.020**

*The variables “regions of the country” and “age” showed weaker associations and were not included in the multivariate regression to increase the precision of the estimates; B: Estimation of regression model parameters; CI: confidence interval; **Multiple linear regression; ***Reference for education levels, †Dichotomous variables, had as reference the absence of the analyzed event.

Source: Research data.

## DISCUSSION

This is a pioneering study in Brazil that portrays HL conditions of adolescents in different regions of the country. In this investigation, higher HL scores were evident in female adolescents, who attended higher education and used medication.

The average HL score of 25.3 points (±5.4) in the studied population was similar to that of international studies using the same instrument. The survey with 650 Chinese adolescents found an average of 26.3 HL points (±5.89)^(6)^; Meanwhile, in Italy, research with 806 university students found an average of 27.4 points (±4.4)^(7)^;among 746 medical students in China, the average was 25.6 points (±4.5)^(8)^. The highest HL mean was reported in Australia in a survey of 120 adolescents with a mean of 28.25 (±6.0)^(21)^.

The research carried out in Belo Horizonte, Brazil^(12)^, with 384 adolescents, using a Pediatric Quality of Life Questionnaire, indicated an adequate level of HL and reported that the best scores in the social and school domains were associated with the best HL scores. Socialization is one of the dimensions of the 6-D model of HL in adolescents, whose core is school^(10)^. This is a space for interaction and social and participatory development of adolescents and represents an important environment for carrying out health-promoting actions.

Schooling is defended as a HL determinant, that is, health and education are closely linked. In the present study, the highest level of education of adolescents (that is, those attending higher education) showed a positive association with HL, a relationship explored in the third D^(10)^. In line with this, experts point out that HL should be an educational objective and should be implemented early in school environments^(22)^.

The inclusion of practices aimed at promoting health in formal education in Brazil is supported by public policies. The School Health Program and the Education Guidelines and Bases Law postulate the obligation to teach health-related topics in Brazilian school curricula^(23)^. Despite this, the implementation of such teaching encounters several barriers such as the disarticulation between health services and schools, the carrying out of actions by professionals outside the school routine and the sporadic nature^(24)^. It is noteworthy that HL can increase health equity and, when encouraged in an educational environment, contribute significantly to the adoption of protective behaviors, as well as to the selection of safe information. These are necessary skills, especially in the face of the infodemic, just as they are of current interest to children and adolescents in the digital world, as portrayed by the sixth dimension (the digital) of the HL theoretical model for these vital cycles^(10)^.

Nursing activities in school environments in Brazil have been reported since 1910, in spaces such as school infirmaries or, more recently, in actions of the School Health Program^(23)^. The presence of nurses in the school routine has considerable potential for constructing interventions focused on the development of HL, the early identification of illnesses and the construction of healthier spaces, in addition to enabling intersectoral engagement, with the participation of an interdisciplinary team, in the development of activities aimed at health education. Furthermore, school spaces can contribute to the training of adolescents involved in health actions in their communities, in accordance with the fifth D of the aforementioned theoretical model^(10)^.

Dependence on intergenerational relationships of knowledge transmission and inequalities, explored in the second and fourth D^(10)^, can contribute to the construction of care habits related to social roles^(10)^. The influence of gender on HL levels is reported in the scientific literature^(25)^; in this study, a positive relationship was observed between being female and having higher levels of HL.

Lower HL scores among males are observed worldwide, being investigated in light of the construction of gender roles. Men have higher rates of morbidity and mortality, prevalence of serious and chronic health conditions, as well as low adherence to care actions^(26)^. In Brazil, the National Men’s Health Policy was enacted in 2008, but advances in assistance to this population are gradual, with actions still poorly structured^(26)^, especially for male adolescents.

Furthermore, adolescents and young males constitute the main risk group for homicide mortality in the Brazilian population^(27)^. A literature review study highlighted the organization of Primary Care as a pillar for the restructuring of care for men, with the development of health promotion actions; The need for access to services stands out, through flexible schedules and continuity of care, through territorialization^(26)^. Furthermore, discussion about health self-management should be encouraged among adolescents and young men; It is believed that these changes can contribute to an increase in HL in this group and vice versa, which consequently can contribute to improving the quality of life in this segment of the population.

Disease patterns and perceptions of the health-disease process, encompassed in the first D of the theoretical framework^(10)^, influence the level of HL^(10)^. A review with meta-analysis recently showed a relationship between a higher HL score and better adherence to drug therapy, especially in patients with chronic diseases^(28)^. The use of medication may have stimulated information-seeking behavior among adolescents, with an impact on HL.

The development of adolescents in the digital era is a characteristic of a large proportion of adolescents around the world^(29)^ and interaction with digital media allows for increased access to information and services that can influence the group’s HL^(30)^. Given this and considering that this research was developed using digital relationship platforms and services, this dimension of the HL of Brazilian adolescents should be deepened and investigated as a strategic resource for health promotion.

The present study presents unprecedented data about the HL of Brazilian adolescents and its relationship with sociodemographic and clinical factors in light of the German theoretical model for children’s HL^(10)^. Limitations of this research include the lack of race and ethnicity, as well as recruitment in a virtual environment, which may have excluded a portion of socially vulnerable Brazilian adolescents without access to the internet, computers or similar devices.

## CONCLUSION

In this study, HL of Brazilian adolescents presented a score similar to the averages found in other countries, and the higher HL scores were shown to be more associated with the female gender, with a higher level of educational background and with the use of medication. The variables that influence HL must be valued in health interventions; Furthermore, cross-cutting issues such as adequate attention to the health of male adolescents, skills and practices related to the appropriate use of medicines and self-management of health must be reinforced in the intersectoral agenda for health promotion in adolescence. Based on the findings of this research, it is recommended that health professionals should work in interdisciplinary teams and in spaces such as schools and universities in activities aimed at developing HL; Such actions require a permanent nature, an early start in the school curriculum and special attention to adolescents, especially for males.
